# Retroviral intasomes search for a target DNA by 1D diffusion which rarely results in integration

**DOI:** 10.1038/ncomms11409

**Published:** 2016-04-25

**Authors:** Nathan D. Jones, Miguel A. Lopez Jr, Jeungphill Hanne, Mitchell B. Peake, Jong-Bong Lee, Richard Fishel, Kristine E. Yoder

**Affiliations:** 1 Department of Molecular Virology, Immunology and Medical Genetics, The Ohio State University Medical Center, Columbus, Ohio 43210, USA; 2 The Interdisciplinary Biophysics Graduate Program, The Ohio State University, Columbus, Ohio 43210, USA; 3 Department of Physics, Pohang University of Science and Technology (POSTECH), Pohang 790-784, Korea; 4 Interdisciplinary Bioscience and Bioengineering, POSTECH, Pohang 790-784, Korea; 5 Department of Physics, The Ohio State University, Columbus, Ohio 43210, USA

## Abstract

Retroviruses must integrate their linear viral cDNA into the host genome for a productive infection. Integration is catalysed by the retrovirus-encoded integrase (IN), which forms a tetramer or octamer complex with the viral cDNA long terminal repeat (LTR) ends termed an intasome. IN removes two 3′-nucleotides from both LTR ends and catalyses strand transfer of the recessed 3′-hydroxyls into the target DNA separated by 4–6 bp. Host DNA repair restores the resulting 5′-Flap and single-stranded DNA (ssDNA) gap. Here we have used multiple single molecule imaging tools to determine that the prototype foamy virus (PFV) retroviral intasome searches for an integration site by one-dimensional (1D) rotation-coupled diffusion along DNA. Once a target site is identified, the time between PFV strand transfer events is 470 ms. The majority of PFV intasome search events were non-productive. These observations identify new dynamic IN functions and suggest that target site-selection limits retroviral integration.

## Abstract (web summary)

Retroviral Integration into the host DNA is a rare event with evidence suggesting that it relies on DNA topology and sequence. Here the authors present single molecule evidence that shows the retroviral integration machinery vigorously scans the target DNA by 1D-diffusion but infrequently integrates.

## Graphical abstract

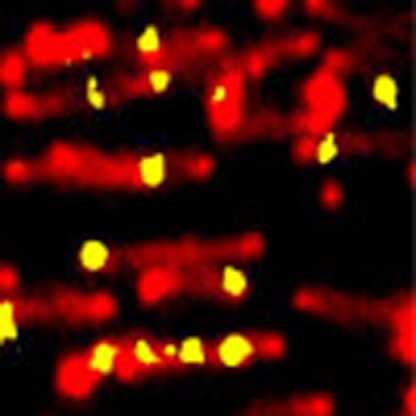

Retroviral integration is infrequent compared with the number of viral complementary DNA reverse transcripts found in infected cells^1^. The possibility that integration chemistry might account for this disparity was underscored by studies that suggested significant time separated the two HIV IN-catalysed strand transfer events^2^. These integration kinetics contrast the time between the murine leukaemia virus (MLV) strand transfer and the resolution of the 5′-Flap and 4 bp gap by the host DNA repair machinery that appeared to occur near simultaneously^3^. In addition, high-throughput sequencing of integration sites has suggested that most retroviruses display a subtle sequence preference^4^,^5^, although whether retroviruses seek out these relatively degenerated nucleotide arrangements is unknown. A preference for distorted DNA^6^,^7^,^8^ was highlighted by structural reports that revealed the prototype foamy virus (PFV) intasome bound to a bent target DNA^9^,^10^ and a related cryo-EM reconstruction that indicated an unusual DNA curvature when it was bound to a nucleosome^11^. Importantly, these foundational studies have been unable to fully resolve the animated progressions that ultimately result in retroviral integration.

Here we utilize single molecule imaging analysis to examine the dynamic properties of the retroviral intasome with a target DNA. We determine that the two strand transfer events associated with PFV integration are separated by 470 ms, which is nearly four orders-of-magnitude faster than that reported for HIV^2^. In addition, we observe vigorous target search processes on linear DNA in which the PFV intasome performs one-dimensional (1D) rotation-coupled diffusion while in continuous contact with the DNA backbone. The target search does not require catalytically active PFV IN suggesting additional functional domains within the PFV intasome that control the target search process. Remarkably, integration is rare compared with the number of target search events. These observations are consistent with the conclusion that target-site selection limits retroviral integration.

## Results

### Strand transfer catalysed by PFV intasomes

**Figure 1:**
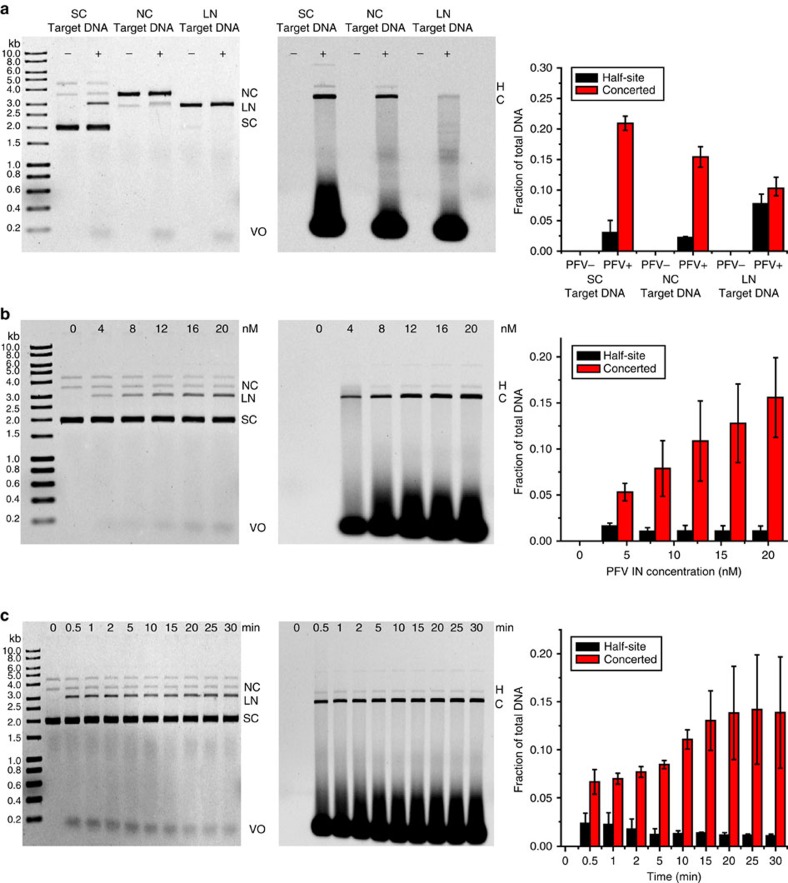
Biochemical analysis of wild type PFV intasome integration. Bulk integration studies into the pMP2 target DNA were performed at ambient temperature (21±1 °C). (Left panels) Ethidium bromide stained gel; (middle panels) Cy5 viral donor emission; (right panels) Quantitative analysis of band intensities. H, half-site integration product; C, concerted integration product. From supercoiled (SC), nicked circular (NC) and linear (LN) target DNAs. For the LN DNA target the half-site products co-migrate with linear target (marked by C), while concerted products migrate as a smear between LN target and viral oligonucleotide DNA (VO). (**a**) Integration of purified Cy5-labelled wild type PFV IN intasome (20 nM) with various target DNA (50 ng) conformations. (**b**) Titration of wild type PFV IN intasome (nM at top) into supercoiled target DNA (50 ng). Note that the fraction of concerted integration product increases with increasing concentration of PFV intasome, while the fraction half-site remained relatively unchanged with increasing PFV intasome. (**c**) Time course (min at top) of wild type PFV IN intasome (20 nM) integration into supercoiled target DNA (50 ng). Error bars indicate s.d. from two independent experiments.

The retroviral intasome is the fundamental nucleoprotein complex that catalyses integration into a target DNA^12^. To examine the dynamics of retroviral integration, we reconstituted PFV intasomes that contained recombinant PFV IN tetramer and two Cy5-labelled oligonucleotides bearing the U5 sequence of the viral cDNA LTR (Supplementary Fig. 1). As expected, the purified PFV intasomes preferred supercoiled DNA as a target (Fig. 1a)^13^ and were capable of catalysing robust integration at ambient temperature (21±1 °C), where the single molecule imaging instruments operate (Fig. 1b,c).

**Figure 2:**
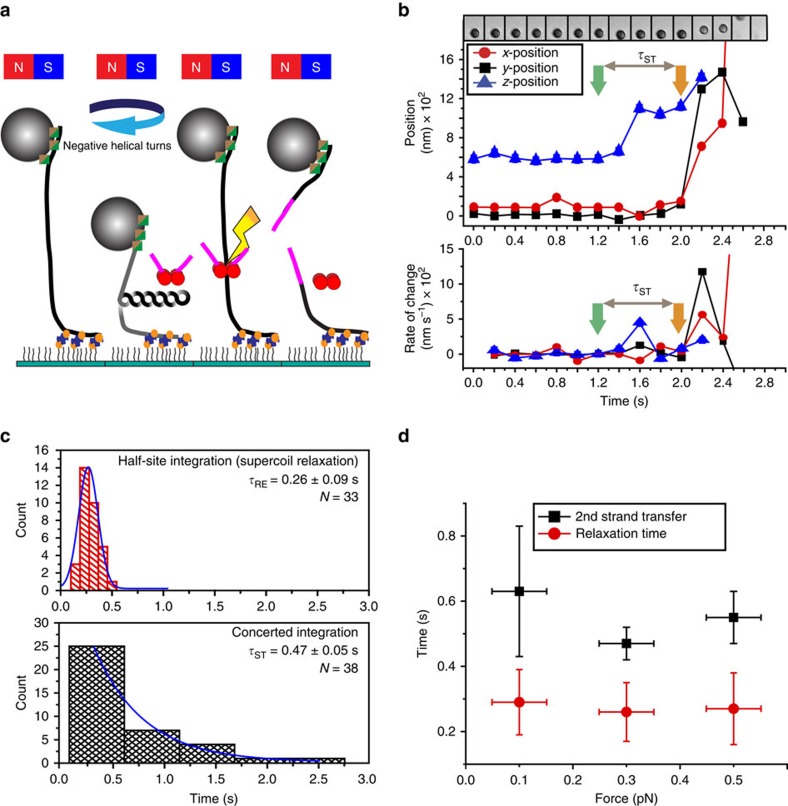
Magnetic tweezers resolves the kinetics of PFV intasome strand transfer. (**a**) Experimental analysis of PFV intasome strand transfer kinetics. DNA bound to an SPM bead and the surface of the flow cell (left) is supercoiled (left centre) by clockwise rotation of ferromagnets under low extension force. PFV intasome interaction with the supercoiled target DNA results in integration where the first strand transfer event creates a strand scission that releases the supercoils (right centre). The second strand transfer event creates a double stand break (DSB) that results in SPM bead dissociation from the target DNA tethered to the surface (right). (**b**) (top panel) A representative SPM bead image during a concerted integration event. (middle panel) A 3D (*x*,*y*,*z*) trace of the SPM bead position. (bottom panel) The rate of SPM bead position change during a concerted integration event. The first strand transfer (green arrow; at 1.2 s) alters the SPM bead height in the *z*-direction as a result of DNA relaxation. The second event (orange arrow; at 2.0 s) alters both the *x*- and *y*-direction, while the *z*-direction could not be clearly resolved because the SPM bead leaves the field of view (see Supplementary Fig. 3a for additional examples). The definition of strand transfer time (*τ*_ST_) is shown. (**c**) The kinetics of DNA relaxation (*τ*_RE_) as a result of one strand transfer event during half-site integration (top panel) and the kinetics of two strand transfer (*τ*_ST_) events as a result of concerted integration. See Methods for the analytical definitions of *τ*_RE_ and *τ*_ST_. The integration analysis shown was performed at a fixed applied force (*F*_*D*_=0.3 pN) on the supercoiled DNA (-10 turns). (**d**) The distribution of relaxation times (*τ*_RE_) and the times between the two strand transfer events (*τ*_ST_) at various force values (Supplementary Fig. 3b). *X*-axis error bars indicate s.e. of force measurements. *Y*-axis error bars indicate s.e. and s.d. of *τ*_RE_ and *τ*_ST_, respectively. The frame rate for smMT was 100–200 ms.

Single molecule magnetic tweezers (smMT) were utilized to determine the kinetic characteristics of two retroviral strand transfer events^14^,^15^,^16^,^17^. In this system, one end of a linear target DNA was attached to an engineered flow cell surface via multiple biotin–NeutrAvidin linkages, while the other end of the DNA was attached to a superparamagnetic (SPM) bead via multiple digoxygenin–antidigoxygenin (dig-antidig) linkages (Fig. 2a). These assorted linkages constrain the DNA preventing rotation of the helical strands around their common axis. Controlled positioning of a permanent magnet relative to the SPM bead may be exploited to apply well-defined extension and rotational forces onto this tethered target DNA (Supplementary Fig. 2)^14^,^17^,^18^. For example, clockwise rotation of the magnet physically introduces linking number changes (*−ΔLk*) in the DNA between the SPM bead and the surface that at low extension forces (0.1–0.5 pN) generates defined negative plectonemic supercoils (*−ΔWr*) by the simple relationship *ΔLk*=*ΔTw*+*ΔWr*, where *Tw* remains relatively constant at ∼10.4 bp per turn^19^,^20^. In the smMT system, these negative plectonemic DNA supercoils reduce the apparent height of the SPM beads relative to the surface (Fig. 2a; Supplementary Fig. 2b)^21^.

The first stand transfer event during a concerted PFV intasome integration in the smMT system introduces an ssDNA scission that will relax the plectonemic supercoils within the tethered target DNA, returning the SPM bead to its initial position (Fig. 2a). Since the viral U5 cDNA oligonucleotides are not connected, the second strand transfer event will introduce a double-stranded break (DSB) into the target DNA releasing the SPM bead from the image plane (Fig. 2a). A three-dimensional (3D) tracking algorithm was used to follow the SPM bead position^22^ that was combined with in-house developed software to analyse *x*, *y* and *z* coordinates and associated force measure. The strand transfer time (*τ*_ST_) was easily visualized and defined as the first detectable SPM bead movement in the *z*-direction (first strand transfer) followed by the first detectable *x*- and/or *y*-movement (second strand transfer) that ultimately released the SPM bead (Fig. 2b; Supplementary Fig. 3a; see Methods for definitions and data analysis). Recombinant PFV intasomes may also perform a single strand transfer event, termed half-site integration (Fig. 1). Half-site integration uniquely introduces an ssDNA scission that only results in *z*-direction movement and may be used to determine a supercoil relaxation time (*τ*_RE_; see Methods for definitions and data analysis). However, half-site integration events are unlikely to have physiological significance since only concerted joining of the two viral cDNA ends yields a functional provirus *in vivo*.

Both concerted and half-site integration events were observed at multiple DNA extension forces (Fig. 2c,d; Supplementary Fig. 3b,c). The *τ*_RE_ for PFV half-site integration was fit to a normal distribution (0.26±0.10 s, at 0.3 pN) that was virtually identical to the *τ*_RE_ induced by the Nt.BspQ1 nicking endonuclease (0.25±0.05 s, at 0.3 pN; Supplementary Fig. 4). These results are consistent with the conclusion that PFV intasome interactions with the target DNA do not affect DNA supercoil relaxation kinetics. The distribution of concerted integration events fit a single exponential decay consistent with a unique *τ*_ST_ between the first and second strand transfer events (0.47±0.05 s at 0.3 pN). Because we define the start of the first strand transfer as well as the start of half-site integration as the point of the first detectable *z*-direction rate change (see Methods for definitions and data analysis), the contribution of *τ*_RE_ to *τ*_ST_ would appear minimal. However, a scenario where supercoil relaxation is required to detect the first strand transfer event could theoretically increase the time between the first and second strand transfer events (*τ*_ST_') for PFV concerted integration to 0.73 s (*τ*_ST_'=*τ*_RE_+*τ*_ST_).

Both *τ*_RE_ and *τ*_ST_ appeared to be independent of DNA extension forces between 0.1 and 0.5 pN (Fig. 2d). There were very few integration events at extension forces above 0.5 pN, where plectonemic supercoils transition to toroidal superhelicity and DNA with altered twist^17^,^21^. The relative frequency of concerted (52%) and half-site (48%) events appeared similar below 0.5 pN (Supplementary Fig. 5a). Moreover, we found that the distribution of integration times for both concerted and half-site events was identical regardless of whether the reaction was carried out for 30 or 90 min (Supplementary Fig. 5b). These observations imply that PFV intasome-catalysed concerted and half-site integrations are intrinsically unrelated and that long incubation times do not drive half-site events into concerted events. We cannot rule out an effect of the low extension force on PFV integration since the frequency of half-site events appeared ∼4-fold greater with the smMT system compared with bulk studies with supercoiled DNA (compare Fig. 1 with Supplementary Fig. 5a). Nevertheless, the sub-second time between the two PFV intasome-catalysed strand transfer events strongly suggests that integration catalysis is unlikely to limit concerted retroviral integration.

### PFV intasomes search the target DNA by 1D diffusion

**Figure 3:**
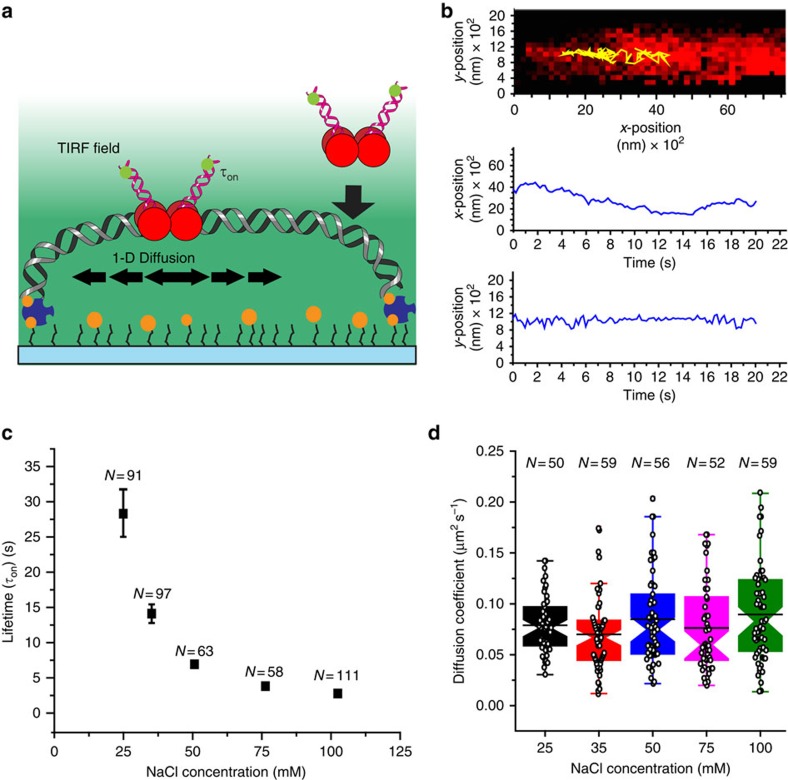
PFV intasomes search a target DNA by 1D diffusion. (**a**) Experimental system for PFV intasome interaction with a target DNA using smTIRF imaging. The total internal reflection fluorescence (TIRF) field extends 200–250 nm into the flow cell (teal background). A target DNA is stretched by hydrodynamic flow and bound at both ends by the biotin–NeutrAvidin over the surface. The PFV intasomes were reconstituted with Cy3-labelled viral U5 cDNA. When the Cy3-labelled PFV intasome interacts with the target DNA inside the TIRF field, the fluorophore is excited allowing visual tracking of particles. (**b**) Representative trace of PFV intasome diffusion (yellow) on a target DNA (red) at a 300-ms frame rate. (**c**) Distribution of lifetimes (±s.e.) for wild type PFV intasomes at different NaCl salt concentrations (Supplementary Fig. 6). (**d**) Box plots of the diffusion coefficients distribution for wild type PFV intasomes at different NaCl salt concentrations at a 100-ms frame rate (Supplementary Fig. 6). Box plots show the mean (indentation), median (black line), upper and lower quartile (box ends), and outliers (whiskers).

The target search before retroviral integration may occur by 3D collision alone, or 3D collision followed by 1D diffusion along the DNA^23^. 1D diffusion is a well-recognized process that significantly enhances the search for sequences, lesions and structures on DNA^24^. We utilized single molecule total internal reflection fluorescence (smTIRF) microscopy to visualize individual PFV intasomes associated with a target DNA^25^,^26^,^27^. A truncated 24 kb λ-based target DNA containing bilateral 5′-biotins was attached at one end to a passivated flow cell surface via PEG–biotin–NeutrAvidin and the DNA gently stretched by defined hydrodynamic flow force to ultimately link the other end via PEG–biotin–NeutrAvidin (Fig. 3a, see Methods)^25^,^26^,^27^.

PFV intasomes were reconstituted with Cy3-labelled U5 viral cDNA (Cy3-PFV; Supplementary Table 1). The substitution of Cy3- for Cy5-labelled U5 viral cDNA enhanced photostability and lifetime in the smTIRF system. These Cy3-PFV intasomes were then introduced into the flow cell, the flow was stopped and the DNA continuously imaged for up to 30 min. We observed single and multiple PFV intasomes that travelled along the target DNA by 1D diffusion (Fig. 3b; Supplementary Movie 1). Individual PFV intasome particles were tracked and the resulting histogram fit to a single exponential decay to determine the lifetime (*τ*_on_=2.1±0.1 s at 100 mM NaCl, *N*=111; Supplementary Fig. 6). While too short to accurately determine above 100 mM, the lifetime showed an inverse correlation with NaCl concentration (Fig. 3c; Supplementary Fig. 6). These observations are consistent with the significant ionic interaction(s) between the PFV intasome and the target DNA.

The *x*- and *y*-positions of Cy3-PFV were followed on the DNA to determine the mean squared displacement (MSD) and a diffusion coefficient (*D*=0.082±0.005 μm^2^ s^−1^ at 100 mM NaCl, *N*=59; Supplementary Fig. 6; see Methods). We found that the MSD in the *y*-direction (<Δ*y*^2^>) resulting from tethered DNA Brownian motions contributed 0.7±0.2% (s.d.; with a maximum of 2%) to the diffusion coefficient determined from the MSD in the *x*-direction alone (<Δ*x*^2^>; Supplementary Fig. 7; see Methods). Together with the lifetime, these observations suggest that the PFV intasome may scan ∼1,600 bp of target DNA before disassociation.

Two fundamentally different 1D search mechanisms have been described, sliding and hopping, that engender different ionic interactions with the DNA^23^,^28^. Diffusion by a sliding mechanism conserves the protein–DNA ionic interactions and is expected to be constant and independent of ionic conditions^23^. In contrast, diffusion by a hopping mechanism must alter protein–DNA ionic interactions with each step and is expected to increase with ionic strength^23^. We found that diffusion of the PFV intasome was constant and independent of ionic strength (*D*_avg_=0.079±0.006 μm^2^ s^−1^; Fig. 3d; Supplementary Fig. 6). While our studies cannot rule out secondary DNA interactions such as inter-segmental transfer^29^, these results are consistent with the conclusion that the PFV intasome diffuses by a sliding mechanism along the DNA.

Theoretical modelling of PFV intasome diffusion based on comparable observations with other DNA binding proteins^30^,^31^ and using the crystal structure to determine intasome dimensions^9^, we calculated a simple sliding diffusion coefficient of ∼45 μm^2^ s^−1^ (Supplementary Note 1)^32^. This is at least two orders-of-magnitude faster than our measured diffusion coefficient. We estimated an average free-energy barrier (*ɛ*) of 1.1±0.2 *k*_B_*T* for rotational sliding of the PFV intasome along DNA (Supplementary Note 1)^30^,^31^. This is significantly less than the 2 *k*_B_*T* energy barrier for biologically relevant sliding with rotation on DNA^33^. Taken as a whole, this evaluation is consistent with the hypothesis that PFV intasomes search a target DNA by 1D rotation-coupled translational diffusion along the DNA.

**Figure 4:**
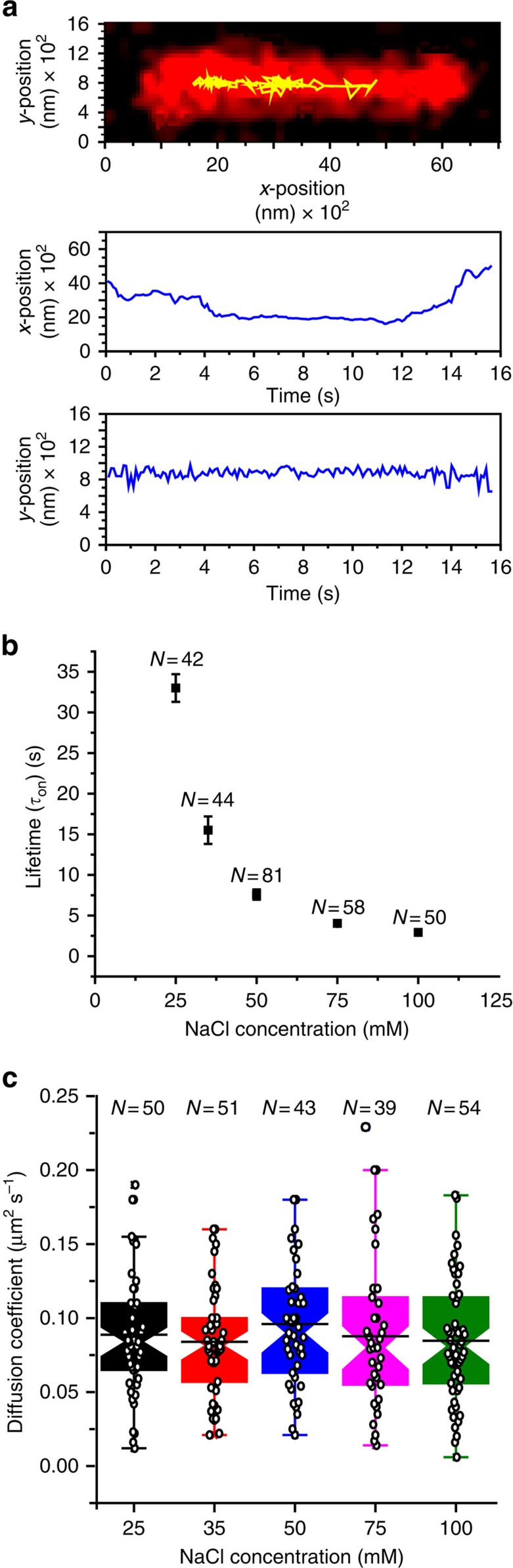
The PFV IN(D128N) intasome search on target DNA. (**a**) Representative trace of PFV IN(D128N) intasome diffusion (yellow) on a target DNA (red) at a 200-ms frame rate. (**b**) Distribution of lifetimes (±s.e.) for PFV IN(D128N) intasomes at different NaCl concentrations at a 200-ms frame rate (Supplementary Fig. 8). (**c**) Box plots of the diffusion coefficients for PFV IN(D128N) intasomes at different NaCl concentrations at a 100-ms frame rate (Supplementary Fig. 8). Box plots show the mean (indentation), median (black line), upper and lower quartile (box ends), and outliers (whiskers).

We prepared catalytically inactive intasomes using PFV IN(D128N) and Cy3-labelled viral U5 cDNA. A similar smTIRF analysis found that the lifetime (2.9±0.3 s), diffusion coefficient (*D*_avg_=0.082±0.031 μm^2^ s^−1^) and diffusion properties were similar to the wild-type PFV intasomes (Fig. 4; Supplementary Fig. 8). These results are consistent with the conclusion that altering the PFV intasome catalytic site does not affect the target DNA search process. Taken together, these observations suggest that residues and/or domains outside the catalytically active core are required to accomplish the 1D diffusion target search process.

### Integration is rare compared with the target search

**Figure 5:**
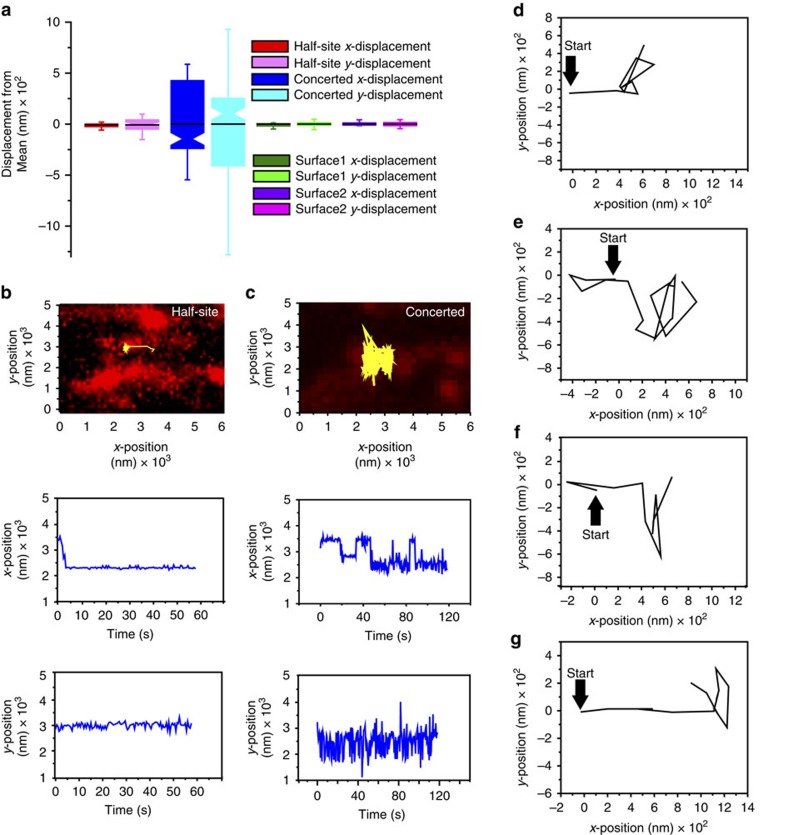
Imaging PFV intasome integration events into linear target DNA using smTIRF. (**a**) Box plots of the frame-by-frame *x*- and *y*-displacement from the mean for (left-to-right): half-site integration example (**b**); the concerted integration example (**c**); and two random particles that were stuck to the flow cell surface (surface 1 and surface 2). Box plots show the mean (indentation), median (black line), upper and lower quartile (box ends), and outliers (whiskers). (**b**) (top) Representative trace of a wild-type PFV intasome (yellow) half-site integration event on DNA (red) at a 500-ms frame rate. (middle) *x*-position and (bottom) *y*-position traces over time. Note reduced *x*-position and increased *y*-position indicative of a half-site integration event where the Cy3-labelled viral U5 cDNA donor is linked to a target DNA molecule attached at both ends and undergoing Brownian movement (see **a**). (**c**) (top) Representative trace of a wild type PFV intasome (yellow) concerted integration event on DNA (red) at a 500-ms frame rate. (middle) *x*-position and (bottom) *y*-position traces over time. Note large *x*- and *y*-position Brownian movement indicative of a broken DNA attached at one end following a concerted integration event (see **a**). (**d**–**g**) Initial frame-by-frame traces of the four observed concerted integration events. (**d**) The initial tracking frames for the integration event shown in **c**. All tracking traces (500 ms frame rate) show an initial sliding diffusion for 1–2 s that was followed by a large movement in the *x*-direction (97–236 nm); ultimately resulting in a particle that randomly moved in both the *x*- and *y*-direction consistent with a broken DNA containing a Cy3 label.

Remarkably, only four potential concerted and three potential half-site integration events were observed in 812 recorded target DNA search events (Fig. 5). Integration events could be distinguished from surface-bound fluorophores by their unusual *x*- and *y*-direction motions (Fig. 5a). For example, probable half-site integration events clearly overlaid a Sytox-stained DNA and were associated with a Cy3-PFV intasome that initially underwent *x*-direction diffusion, which was followed by localized *x*- and *y*-direction movement attributable to Brownian motion of the Cy3-labelled viral U5 cDNA attached to a target DNA tethered to the surface at both ends (Fig. 5a,b; Supplementary Movie 2). In contrast, none of the potential concerted integration events were correlated with significant Sytox DNA staining since the broken target DNA is expected to coil near the tether site^34^ (Fig. 5c). Instead, a short *x*-direction diffusion of the Cy3-PFV intasome was followed by a relatively dramatic *x*-direction movement that we interpret reflects the broken DNA contracting to one tether site, with the attached Cy3-labelled viral U5 DNA ultimately displaying *x*- and *y*-direction Brownian motions consistent with random coiled DNA tethered at one end (Fig. 5d–g; Supplementary Movie 3; note that the tracked particle in Fig. 5c,d and Supplementary Movie 3 are the same event). There may be two contributors to the reduced frequency of integration events: (1) the necessary use of a linear DNA that is intrinsically a less-efficient target substrate (Fig. 1) (ref. 2) and/or (2) the resulting broken integration product consisting of coiled target DNA might extended out of the TIRF field making them invisible in the smTIRF system. To further consider this latter possibility, we calculate that 2–4 kb of coiled DNA from either tethered end (20–30% of the total target DNA) may remain continuously in the 200–250 nm TIRF field generated by our smTIRF system^35^. This implies that we may be missing 70–80% of the concerted integration events, which could increase the total to between 15 and 20 out of the 812 recorded target DNA search events. However, even including obscured integrations it is clear that >97% of the PFV intasome search events appear to be nonproductive. It is important to note that even though our recorded events likely reflect half-site and concerted integrations, they are not statistically significant to draw mechanistic conclusions.

## Discussion

To our knowledge, these studies represent the first single molecule imaging analysis of retroviral integration. The results suggest that the two PFV IN-catalysed strand transfer events are discernible but also nearly simultaneous, an observation that significantly contrasts previous bulk HIV-1 integration analysis^2^. In addition, we found that concerted and half-site PFV integration events are intrinsically unrelated, while previous HIV studies suggested that half-site events progress to concerted events^2^. These results appear to reflect previously unrecognized differences between the PFV and HIV integration processes.

There are ∼100 predicted PFV integrase preferred-sequences in the *λ* target DNA used in the smTIRF analysis^13^,^36^. Based on the numbers of observable target molecules in the smTIRF system, we calculate that PFV intasomes should have encountered a preferred target sequence ∼100,000 times during our observation period. The exceedingly low number of integration events suggests that a preferred sequence search on a target DNA by retroviral intasomes is at the very least inefficient. Another possibility is that PFV intasomes search for DNA conformations or structures^6^,^7^,^8^. Consistent with this hypothesis we find that supercoiled DNA is a preferred target^21^. However, it should be noted that there is only a 2–5-fold increase in the PFV intasome integration frequency into supercoiled DNA compared with linear DNA (Fig. 1). These observations suggest that even for a preferred target only 10–15% of search events will be productive. Taken as a whole, we conclude that target site selection limits PFV integration. Finally, the retroviral search mechanics on chromatin is likely to be significantly different since rotation-coupled 1D diffusion is physically limited and host factors may tether the intasome to a nucleosome target^37^,^38^.

## Methods

### Chemicals and enzymes

All chemicals were ultrapure and purchased from Sigma Aldrich (St Louis, MO) or Amresco (Cleveland, OH). Restriction enzymes and T4 DNA ligase were purchased from New England Biolabs (NEB, Beverly, MA) or ThermoFisher Scientific (Waltham, MA). Recombinant PFV IN and catalytically inactive PFV IN(D128N) containing N-terminal His_6_-tag (HHHHHH), thrombin protease site (LVPRGS), HRV3C protease site (LEVLFQGP) and spacers (MGSS-HHHHHH-SSG-LVPRGS-HS-LEVLFQGP-G-PFV IN coding sequence minus PFV N-terminal Met) was purified by a modified method^12^. Briefly, the IN protein was overexpressed in *Escherichiacoli* Rosetta (DE3) that were lysed by sonication and soluble material separated from cellular debris by ultracentrifugation. The cell lysate in 50 mM Tris-HCl (pH 7.5), 500 mM NaCl was subjected to Ni-NTA chromatography, IN protein fractions pooled and the majority of the N-terminal tag removed with in-house purified HRV3C protease (leaving GP-G-PFV IN coding sequence). The protease-treated protein was diluted fourfold with 50 mM Tris-HCL (pH 7.5), 5 mM DTT, 0.1 mM EDTA (Buffer A) loaded onto a Heparin column, washed with Buffer A plus 200 mM NaCl and stepped off with Buffer A plus 1 M NaCl. Highly concentrated protein samples were dialysed into storage buffer containing 50 mM Tris pH 7.5, 500 mM NaCl, 5 mM DTT and 10% glycerol, snap frozen in LN2 and were stable at −80 °C for several months.

Intasomes were assembled by combining highly concentrated PFV IN (120 μM final) with annealed oligomers mimicking pre-processed PFV U5 viral cDNA ends (50 μM final) in 50 mM Bis-Tris Propane-HCl 7.45, 500 mM NaCl and then dialysed overnight at 18 °C against 20 mM Bis-Tris Propane-HCl 7.45, 200 mM NaCl, 2 mM DTT, 25 μM ZnCl_2_. Assembled intasome complexes were separated from aggregates and non-intasome material by Superose 12 10/30 GL size exclusion chromatography in 20 mM Bis-Tris Propane-HCl (pH 7.45), 320 mM NaCl, 50 μM ZnCl_2_, 10 mM DTT and 10% glycerol (Supplementary Fig. 1). Fractions were snap frozen and stable at −80 °C for several months.

### Bulk PFV IN strand transfer analysis

PFV IN intasome catalysed strand transfer into supercoiled, nicked or linear pMP2 target DNA (50 ng) was examined over time at ambient temperature (21±1 °C). The 15-μl reactions included PFV intasome (20 nM) in Buffer A (10 mM Bis-Tris Propane-HCl (pH 7.45), 110 mM NaCl, 5 mM MgSO_4_, 4 μM ZnCl_2_ and 10 mM DTT). Following incubation, the reactions were stopped with the addition of 0.45% SDS and 900 μg ml^−1^ proteinase K. Products were separated on a 1% agarose gel, stained with ethidium bromide (0.1 μg ml^−1^), scanned using a Typhoon 9410 variable mode fluorescent imager (GE Healthcare Life Sciences) and analysed with ImageQuant TL software.

### Magnetic tweezers

Flow cells were engineered with glass cover slides that were affixed with double-sided tape to an aluminium foundation that maximized SPM bead imaging. Prior to attachment, the glass slides were treated with (3-aminopropyl) triethoxysilane followed by a 1:100 mixture of biotin–PEG SVA to mPEG-SVA (Invitrogen). Plasmid pET-29a was digested with EcoRI and SphI yielding a 4,967 bp linear fragment. Biotin and digoxigenin labelled 1 kb linkers were generated by PCR and included a 1:10 ratio of biotin- or dig-labelled dUTP to dTTP in the reaction. The 1-kb PCR products were digested with EcoRI and SphI, ligated to the pET29a fragment with T4 DNA ligase and the linear 7 kb DNA ligation fragment was excised from a low-melt agarose gel stained with ethidium bromide and purified using a QIAquick Gel Extraction kit (Qiagen), precipitated with isopropanol and washed with ethanol to fully remove any remnant ethidium bromide prior to aqueous resuspension. NeutrAvidin (500 μm, Invitrogen) was injected in the flow cell at a rate of 8 μl min^−1^, followed by the 7-kb ligation fragment in the presence of T4 DNA ligase to seal any remnant strand scissions. Tosylactivated M-280 SPM Dynabeads (ThermoFisher Scientific) were coated with anti-digoxigenin antibodies and injected into the flow cell containing the 7-kb DNA ligation fragment that was bound to the surface via a biotin–NeutrAvidin linkage; at 8 μl min^−1^ while agitating the system. The bound DNA was washed extensively with Buffer A to remove T4 DNA ligase and free SPM beads prior to PFV integration analysis. Force extension and introduction of supercoils used two 1 cm^3^ rare earth magnets (Neodynium, Magcraft). For PFV integration analysis, the DNA was wound clockwise with 10 complete turns of the magnets to induce negative supercoils. The SPM beads were imaged using a 530-nm LED lamp (Thorlabs), a × 40 Olympus oil immersion objective and images collected on a 1,024 × 1,024 pixel charge coupled device camera (Grasshopper Express 1.0 MP Mono FireWire 1394b) at a frame rate of 100–200 ms for at least 1,800 s.

SPM bead analysis was performed with the 3D bead tracking software Video Spot Tracker (CISMM at UNC-CH). The time establishing SPM bead movement in any one of the three-dimensions (*x*,*y*,*z*) was determined as the point of departure from zero of the first-derivative of the position versus time curve. The start of a concerted PFV intasome strand transfer event that also includes initial aspects of the supercoil DNA relaxation time was defined as the point of departure from zero of the first-derivative of *z*-position changes versus time curve, while the second strand transfer event was defined as point of departure from zero of the first-derivative of subsequent *x*- or *y*-position changes versus time curve. The strand transfer time (*τ*_ST_) is defined as the time between the first strand transfer and the second strand transfer events and were fit to a single exponential decay to determine the mean lifetime with the standard error (s.e.) of curve fitting. Half-site integration events uniquely alter the *z*-position allowing a clear determination of the supercoil relaxation time (*τ*_RE_), which was defined as the time between the point of departure from zero of the first-derivative of *z*-position change versus time curve to the point that the first-derivative departs from zero again. Distributions for *τ*_RE_ and *τ*_ST_ events were binned using: [number of bins 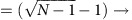 round up]; and 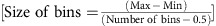, where: Max=maximum value of events; Min=minimum value of events; Bin Start=Min; Bin End=(bin size × number of bins)^39^,^40^. We used 100 ms as the bin minimum for *τ*_RE_ because the data was collected with both 100 and 200 ms frame rates. The *τ*_RE_ events were fit to a Gaussian distribution to determine the mean and s.d. from the mean. All fittings were done with Origin software (OriginLabs, Northampton, MA).

### Single molecule tracking on a target DNA

A *λ*-based target DNA was prepared by digestion with either AvrII (New England Biolabs) or isoschizomer XmaJI (ThermoFisher Scientific) yielding 24.3 and 24.1 kb fragments with cosL-AvrII and AvrII-cosR ends, respectively. Annealed AvrII and cosL linker DNAs were first added to the *λ* fragments with T4 DNA ligase (Supplementary Table 1). Excess linker DNAs were removed by Amicon Ultra-4 centrifugal filters (100 kDa cutoff; Amicon) followed by the addition of the annealed cosR linker DNA in a new T4 DNA ligase reaction. The resulting 24 kb DNA products were excised from a low-melt agarose gel stained with ethidium bromide, treated with β-agarase (NEB), precipitated with isopropanol and washed with ethanol to fully remove any remnant ethidium bromide prior to aqueous resuspension. Engineered flow cells manufactured with quartz slides were treated with (3-aminopropyl) triethoxysilane followed by a 1:300 mixture of biotin–PEG–SVA:mPEG–SVA (Invitrogen)^41^. Neutravidin (500 μm, Invitrogen) was injected in the flow cell prior to the addition of DNA. The *λ*-based target DNA was introduced into the flow cell at 100 μl min^−1^, where stochastic attachment of the 5′-biotin ends to the NeutrAvidin-coated surface under these laminar flow conditions results in fully stretched DNAs linked to the surface at both ends^41^. Purified PFV intasome (0.5 nM) containing Cy3-labelled viral U5 cDNA oligonucleotides was introduced at 10 μl min^−1^, the flow stopped and the Cy3-PFV particles associated with the target DNA were excited with a 532-nm DPSS laser (crystal laser, 50 mW). The Cy3-PFV emission was detected in a prism-type TIRF microscope system (Olympus IX-71, water-type × 60 objective, N/A=1.2; extended with a × 1.6 magnifier) and recorded on an electron-multiplying charge-coupled device camera (EMCCD, Princeton Instruments, ProEM 512 excelon)^41^,^42^.

Lifetime and diffusion tracking measures with the Cy3-labelled wild type PFV intasomes were followed for 1,650 s at 300 ms and 230 s at 100 ms frame rate, respectively. Lifetime and diffusion tracking measures with the Cy3-labelled PFV IN(D128N) intasomes were followed for 230 s at 200 and 100 ms frame rate, respectively. To detect integration events wild-type PFV intasome were followed for 3,600 s at 500 ms frame rate. Following tracking measurements, Sytox orange (100 nM) was introduced into the flow cell to visualize the *λ* DNA. These static images were overlaid onto the tracking movies to identify PFV intasomes that were clearly associated with the target DNA. Particles were tracked using DiaTrack software (Sydney, Australia). Lifetime measures were binned (see above), fit to a single exponential decay and the time±s.e. resolved. The *x*- and *y*-direction tracking information was obtained (DiaTrack) and plotted with Origin software. MSD was determined for single particles at every imaged time point as the square of the diffusion distance in the *x*-direction compared with the preceding time point (<Δ*x*^2^>) (ref. 43). The MSD versus time was displayed for all particle tracks and a linear fit was applied to the first 10% of the data with five frames being the minimum fit requirements to obtain <Δ*x*^2^>/*t* (ref. 43). The diffusion coefficient (*D*) was calculated using the equation: <Δ*x*^2^>=*n* × *D* × *t*, where *n* is the dimensionality constant equal to 2 for linear diffusion^43^. Diffusion coefficients were binned (see above) and fit to a Gaussian distribution with the mean and s.d. from the mean displayed. Combining *x*- and *y*-displacement into the MSD calculation (<Δ*x*^2^>+<Δ*y*^2^>=<Δ*r*^2^>) and using <Δ*r*^2^> to calculate a diffusion coefficient resulted in an average a 0.7±0.2% (s.d.; with a maximum of 2%) difference between the diffusion coefficient calculated using <Δ*x*^2^> alone for MSD. Diffusion coefficients were compiled into box and whisker plots. All fittings and box plots were done with Origin software (OriginLabs).

## Additional information

**How to cite this article:** Jones, N. D. *et al*. Retroviral intasomes search for a target DNA by 1D diffusion which rarely results in integration. *Nat. Commun.* 7:11409 doi: 10.1038/ncomms11409 (2016).

## Supplementary Material

Supplementary Information Supplementary Figure 1-8, Supplementary Table 1, Supplementary Note 1 and Supplementary References

Supplementary Movie 1 Representative movie of PFV wild type intasomes (green) searching model linear target DNAs (red). Scale bar (white) is 4 μm. The frame rate (300 ms) is in real time. (619 frames total)

Supplementary Movie 2 Representative movie of a half-site integration event by a PFV wild type intasome (green). The molecule enters the movie a frame 17 and diffuses in the x-direction until frame 23 before halting and exhibiting the x- and y-direction diffusion characteristic of a point on a DNA that is tethered on both ends. Scale bar (white) is 2 μm. The frame rate (500 ms) is in real-time and particle tracking preformed with DiaTrack software (see Methods). (139 frames total)

Supplementary Movie 3 Representative movie of a concerted integration event by the PFV wild type intasome (green). The molecule enters the movie at frame 33, diffuses in the x-direction until frame 37 where integration occurs (see Fig. S10a for initial frame tracks). The broken DNA then displays Brownian motion consistent with a random coiled duplex tethered at one end. Scale bar (white) is 2 μm. The frame rate (500 ms frame rate) is in real-time and particle tracking preformed with DiaTrack software (see Methods). (266 frames total)
