# Supplementary material for: Retroviral intasomes search for a target DNA by 1D diffusion which rarely results in integration
Source: Nat Commun. 2016 Apr 25;7:11409. doi: 10.1038/ncomms11409 (PMC4848512; doi:10.1038/ncomms11409)
Supplement: Supplementary Information — Supplementary Figure 1-8, Supplementary Table 1, Supplementary Note 1 and Supplementary References [file ncomms11409-s1.pdf]

# Supplementary Figure 1

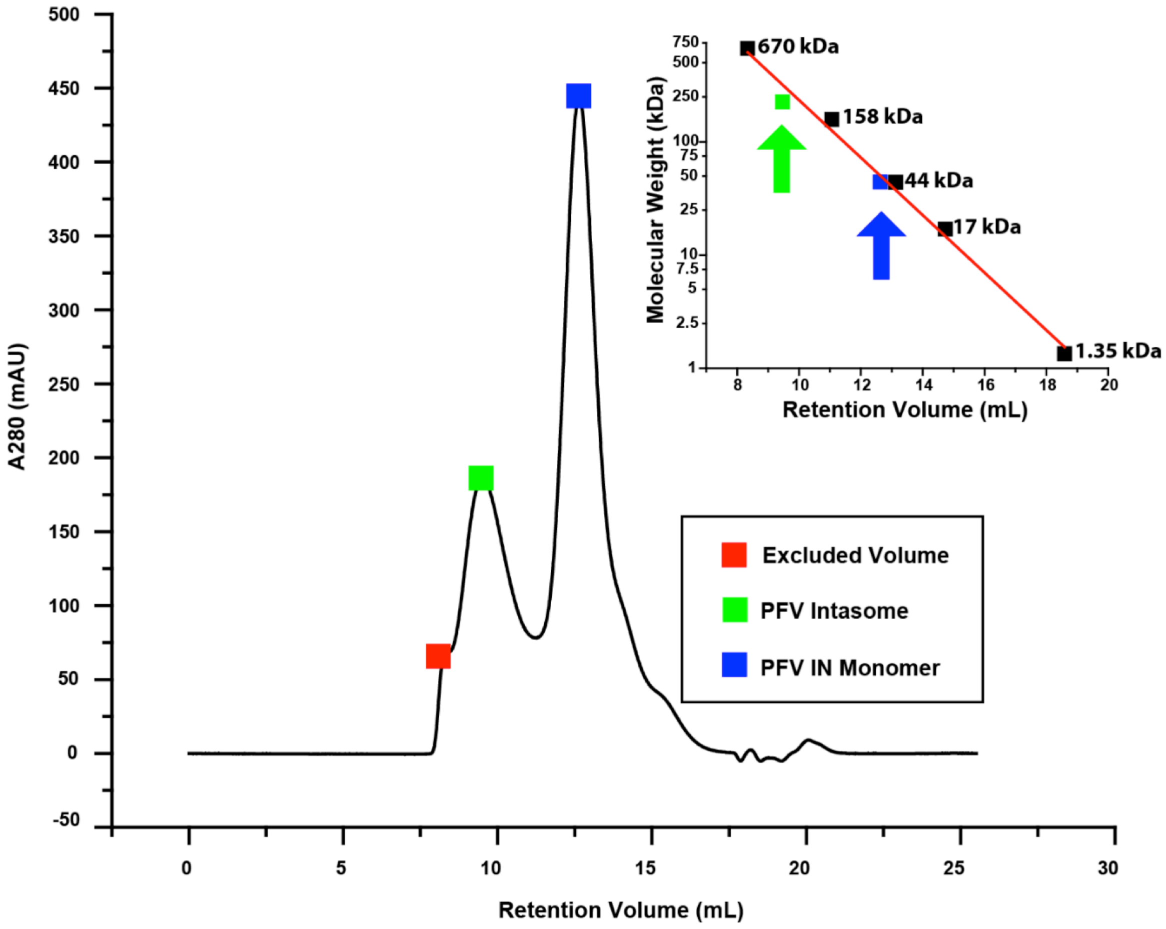


**Supplementary Figure 1. PFV IN Intasome Purification.** Representative elution profile of reconstituted PFV intasome using Superose 12 size exclusion chromatography. Peak locations corresponding to the excluded material including protein aggregates (red), reconstituted PFV intasome (green), and unassembled monomer (blue) are shown in the chromatography trace. (inset) Calibration curve of globular protein molecular weight versus retention volume. Arrows and squares indicate measured retention volume of reconstituted PFV intasome (green) and free PFV IN monomer (blue) on the calibration curve. The difference between the calculated (225.52 kD) and measured (304.5 kD) molecular weight of the PFV intasome is likely a result of the sizeable asymmetry observed in the structural analysis [2].

# Supplementary Figure 2

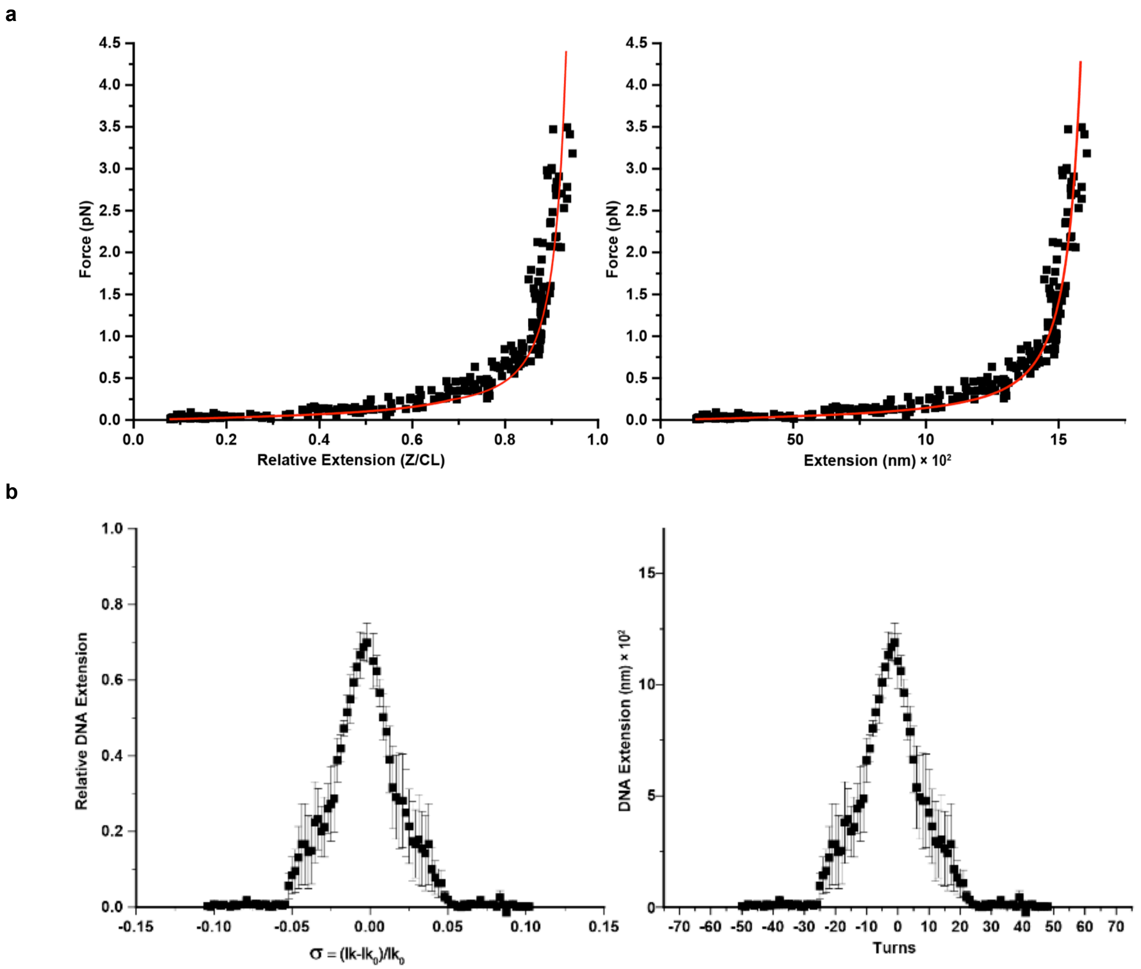


**Supplementary Figure 2. Single Molecule Magnetic Tweezers. a)** Force versus extension curves for double stranded DNA tethered by both helical strands at the surface by multiple biotin-neutravidin linkages and to a superparamagnetic bead by multiple digoxigenin-antidigoxigenin linkages. Relative extension was measured as the stretched length of DNA over the contour length of DNA. The graphs are fit with the worm-like chain model of DNA extension with a contour length of 1,720 nm and a persistence length of 51 nm. **b)** Ferromagnet rotations versus DNA extension for double strand DNA tethered at the surface and to a superparamagnetic bead as in (a). $\sigma$ reflects the excess linking number of rotations. N=10 DNA (s.d.).

# Supplementary Figure 3

a

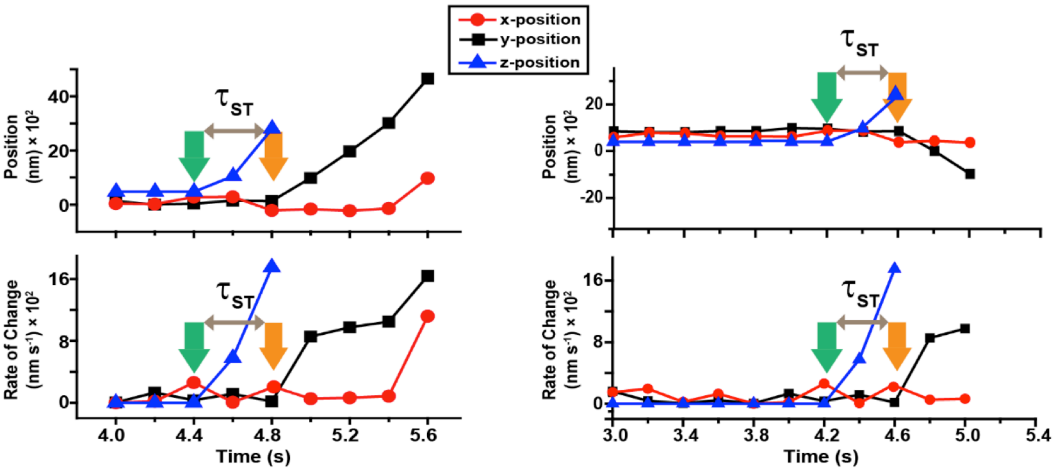


b

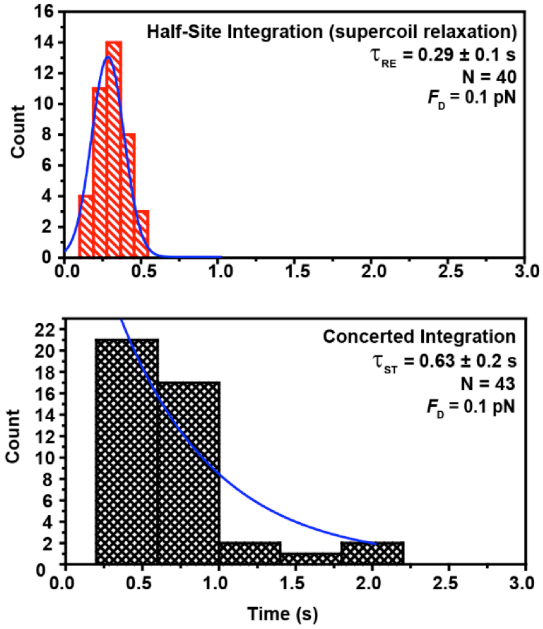


c

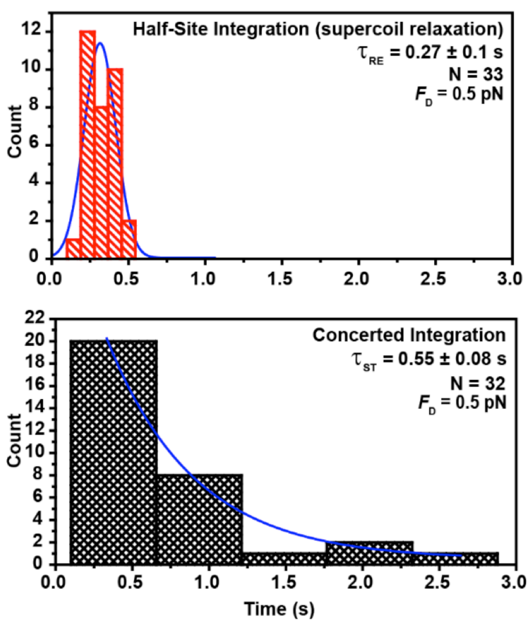


**Supplementary Figure 3. Integration of *wild type* PFV IN Intasome into smMT Tethered DNA. a)** Representative examples of two separate concerted integration events. Note that integration starts with pronounced movement in the z-direction (supercoil relaxation) followed by movement outside of viewing area in both x- and y-directions (SPM bead release). The green arrow indicates the first detectable movement of the SPM bead in the z-direction associated with the first strand transfer event and DNA supercoil relaxation. The orange arrow indicates the first detectable movement in the x- or y-direction associated with the second strand transfer and SPM bead release. **b)** (top) kinetics of DNA supercoil relaxation ($\tau_{RE}$) following half-site integration (N = 40), and (bottom) the time between the first and second strand transfer event ($\tau_{ST}$) during concerted integration (N = 43) at a fixed applied force ($F_D$ = 0.1 pN) on a supercoiled DNA (-10 turns). **c)** (top) kinetics of DNA supercoil relaxation ($\tau_{RE}$) following half-site integration (N = 40) and (bottom) the time between the first and second strand transfer event ($\tau_{ST}$) during concerted integration (N = 43) at a fixed applied force ($F_D$ = 0.5 pN) on a supercoiled DNA (-10 turns). See Methods for definitions and analysis of $\tau_{RE}$ and $\tau_{ST}$.

# Supplementary Figure 4

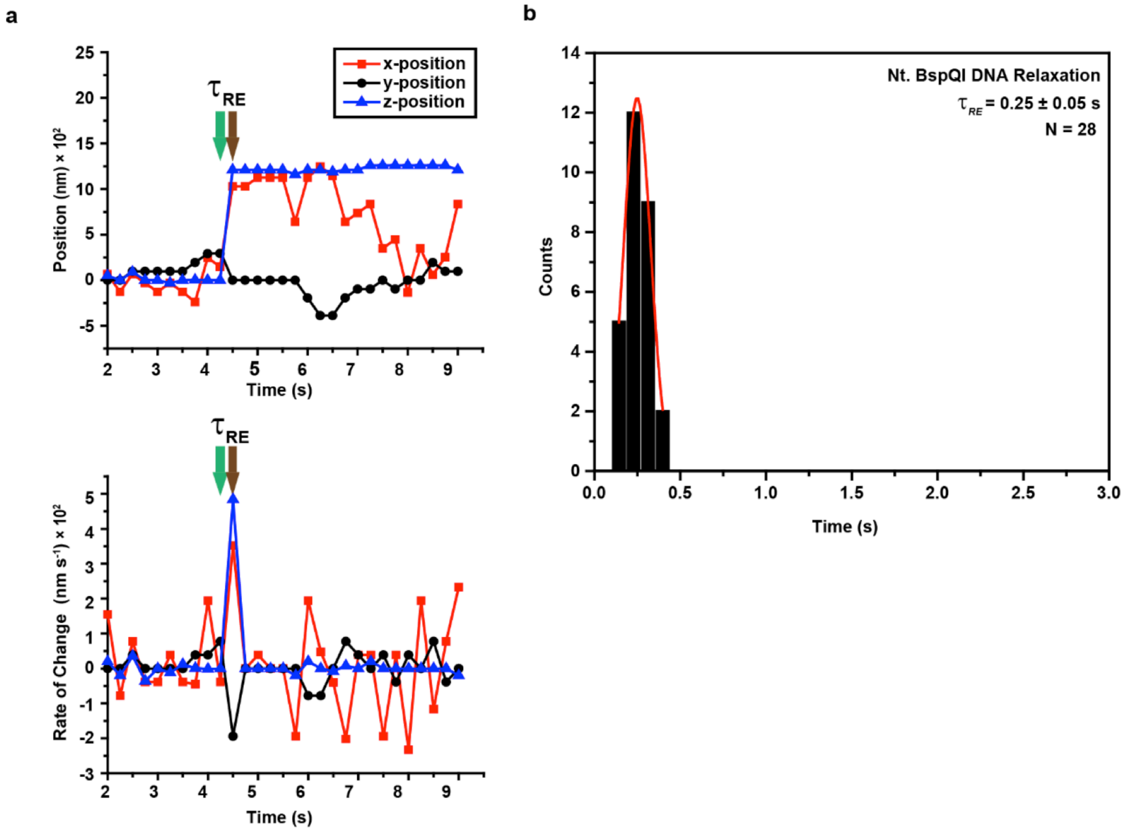


**Supplementary Figure 4. DNA Superhelical Relaxation Catalyzed by Nt.BspQ1. a)** Representative trace in three dimensions (x,y,z) of a DNA superhelical relaxation event induced by the Nt.BspQ1 nickase tracked by SPM bead changes in the smMT system. The DNA was negatively supercoiled (-10 turns) at $F_D$ = 0.3 pN. The green arrow indicates the point of the first detectable movement of the SPM bead in the z-direction associated with the Nt.BspQ1 strand scission initiating DNA supercoil relaxation. The brown arrow indicates point of the last detectable movement in the z-direction associated with DNA supercoil relaxation. The time between these two SPM bead movements is defined as the DNA relaxation time ($\tau_{RE}$). **b)** The distribution of DNA relaxation times catalyzed by a Nt. BspQI strand scission on supercoiled DNA by smMT.

# Supplementary Figure 5

a

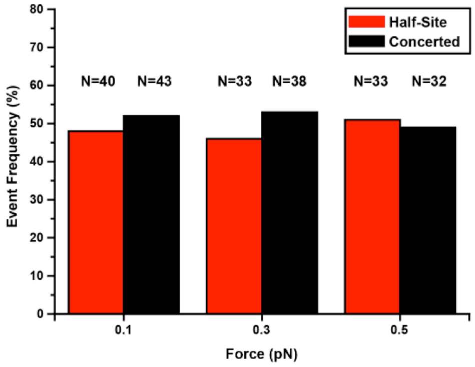


b

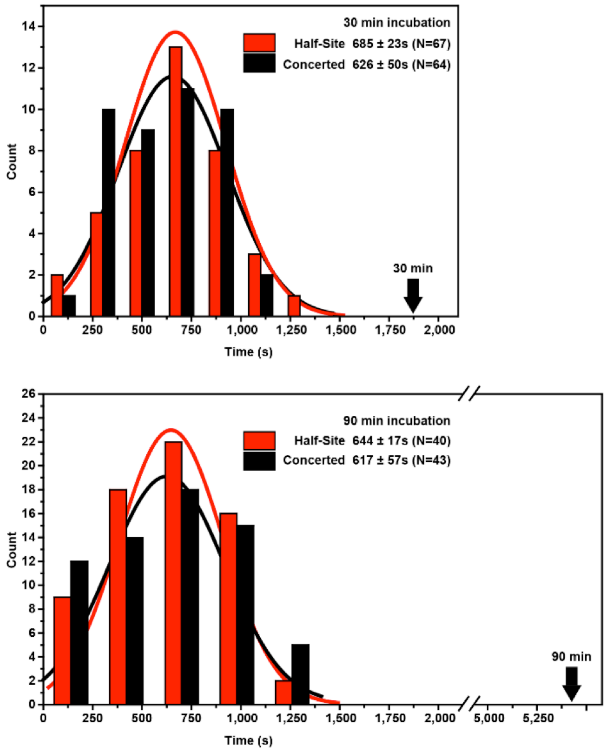


**Supplementary Figure 5. The Distribution of PFV Intasome Integration. a)** The frequency of half-site and concerted integration events (%) observed at 0.1, 0.3 and 0.5 pN. N = number of events.
**b)** Distribution of the time of integration. Time was recorded starting from the moment of PFV intasome injection into the flow cell. Integration events were recorded over a 30 or 90 min period. The binned integration time for half-site integration (red) and concerted integration (black) events are shown and were fit to a Gaussian distribution to derive the mean half-site integration and concerted integration times. N = total number of events.

# Supplementary Figure 6

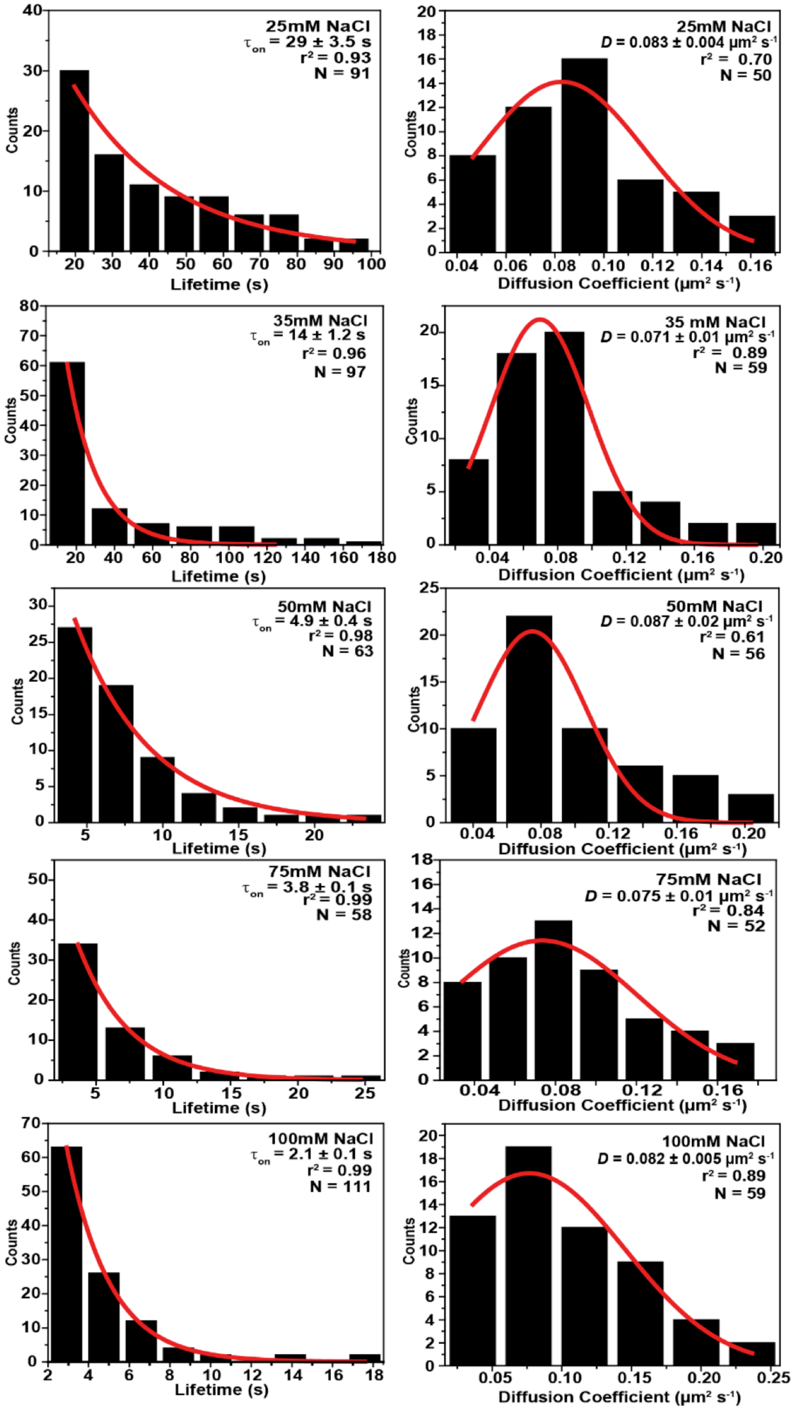


**Supplementary Figure 6. *Wild type* PFV Intasome Lifetime and Diffusion Coefficient on DNA. (Left Panels)** Distribution of the lifetimes ($\tau_{on}$) of searches on linear λ target DNA (see insets for descriptions). **(Right Panels)** Distribution of the Diffusion coefficients ($D$) determined from searches on linear λ target DNA (see insets for descriptions).

# Supplementary Figure 7

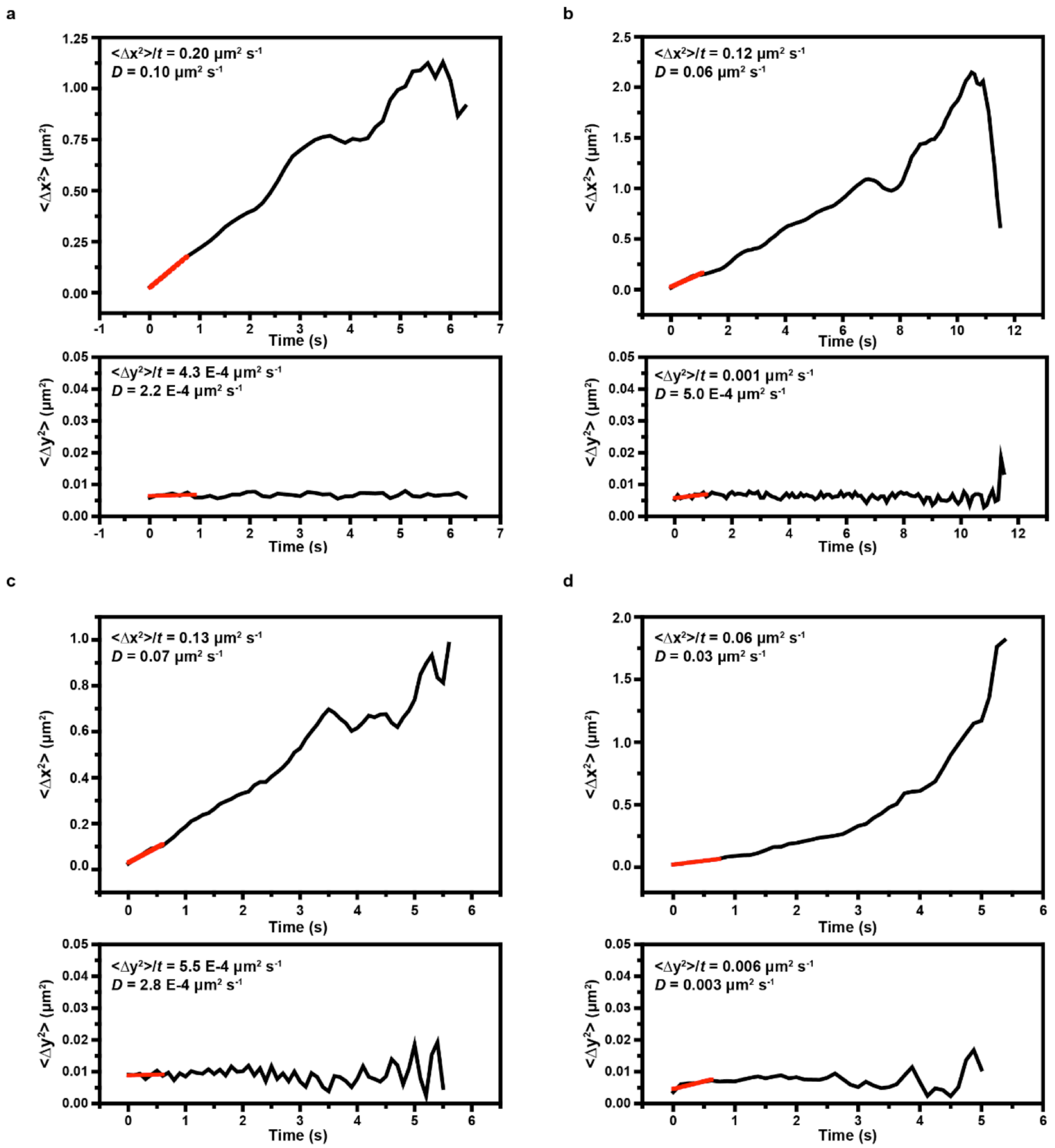


**Supplementary Figure 7*. Wild type* PFV Intasome Diffusion on a Target DNA. (A-D)** Representative trace of mean square displacement (MSD) versus time for four randomly selected PFV intasome molecules associated with their respective target DNAs at 100 ms frame rate. In each panel the $<\Delta x^2>$ (above) and $<\Delta y^2>$ (below) MSD are shown. Linear fits (in red) are applied to the first ten percent of the data with five time points being the minimum fit requirements. Diffusion coefficient ($D$) is calculated by using the diffusion over distance equation ***<MSD> = n*D*t,*** where $n$ is the dimensionality constant. For linear diffusion the dimensionality constant is 2. Note the scale difference between $<\Delta x^2>$ and $<\Delta y^2>$ (see Methods for contribution of $<\Delta y^2>$ to Diffusion coefficient).

# Supplementary Figure 8

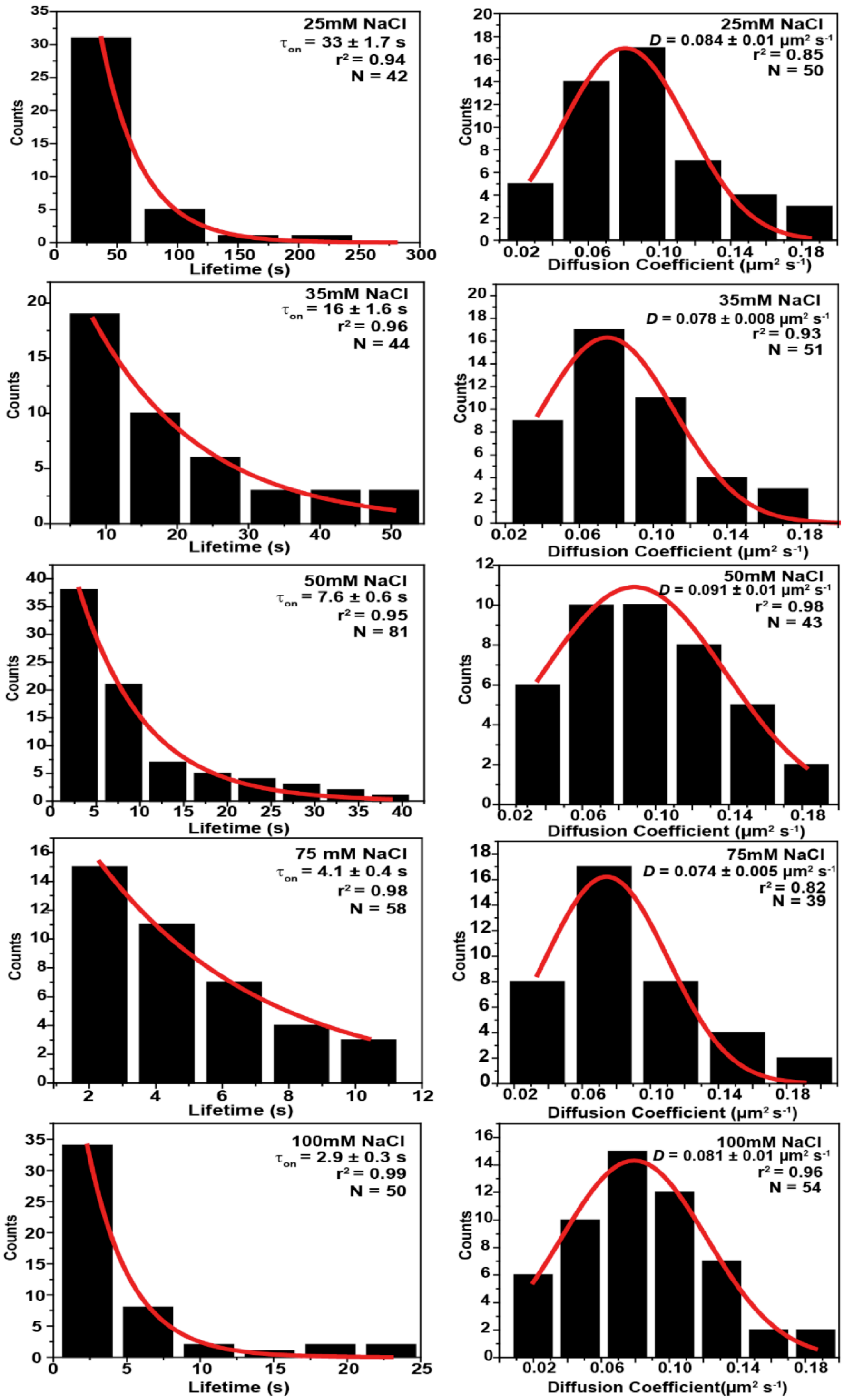


**Supplementary Figure 8. PFV IN (D128N) Intasome Lifetime and Diffusion Coefficient on DNA. (Left Panels)** Distribution of the lifetimes ($\tau_{on}$) of searches on linear λ target DNA (see insets for descriptions). **(Right Panels)** Distribution of the Diffusion coefficients (*D*) determined from searches on linear λ target DNA (see insets for descriptions).

**Supplementary Table 1. Oligonucleotide Sequences**

| Oligonucleotide Name | Sequence (5' → 3') |
|---|---|
| PFV U5-top | ATT GTC ATG GAA TTT TGT ATA TTG AGT GGC GCC CGA ACA G |
| PFV U5-bottom | CTG TTC GGG CGC CAC TCA ATA TAC AAA ATT CCA TGA CA |
| Cy3-U5-bottom | 5'-Cy3-CTG TTC GGG CGC CAC TCA ATA TAC AAA ATT CCA TGA CA |
| Cy5-U5-bottom | 5'-Cy5-CTG TTC GGG CGC CAC TCA ATA TAC AAA ATT CCA TGA CA |
| cosL-linker top | 5'-Biotin-TTT TTA TTG TCC AAC TTG CTG TCC |
| cosL-linker bottom | 5'-phosphate-AGG TCG CCG CCC GGA CAG CAA GTT GGA CAA TAA |
| cosR-linker top | 5'-Biotin-TTT CAC GAC TGA TGA ATT CTA ATG |
| cosR-linker bottom | 5'-phosphate-GGG CGG CGA CCT CAT TAG AAT TCA TCA GTC GTG |
| AvrII-linker top | 5'-Biotin-TTT TAG CGC TAC CCG AAT CAT TCC |
| AvrII-linker bottom | 5'-phosphate-CTA GGG AAT GAT TCG GGT AGC GCT A |

**Supplementary Note 1**. **Modeling of PFV Intasome Diffusion.** The diffusion of a spherical particle through a medium is proportional to the inverse of the drag coefficient $\xi$. Using the drag force for a spherical particle of radius *R* defined by Stokes-Einstein [1], a diffusion coefficient (*D*) may be calculated from:

$$D = \frac{k_B T}{\xi_{Trans}} = \frac{k_B T}{6\pi\eta R} \qquad \text{Eq.1}$$

where:

$k_B$ = Boltzmann Constant.

*T* = Temperature (Kelvin).

$\eta$ = Viscosity of water.

*R* = Stoke's Radius of the complex.

From the PFV IN intasome structure we estimated a Stoke's radius of 5.16 nm [3]. From Eq. 1 we calculate a 1D diffusion coefficient of 42 $\mu m^2\ s^{-1}$. To determine whether PFV intasome diffusion includes rotational as well as translation diffusion we first calculate the free-energy barrier of rotation along the DNA in aqueous solution ($\varepsilon$) [4,5]. These interactions can be described by diffusion on a rugged free-energy landscape (Eq. 2) [4,5].

$$D = b^2 \frac{k_B T}{[6\pi\eta R b^2 + 8\pi\eta R^3 + 6\pi\eta R (R_{OC})^2]} F(\varepsilon) \qquad \text{Eq. 2}$$

Where:

*b* = $(10B_d)/(2\pi)$. Describes the effect of the helical pitch of DNA with regards to sliding.

$B_d$ = Distance between two base pairs (0.34 nm).

$R_{oc}$ = 2.52 nm. Distance between center of mass and the DNA axis.

F($\varepsilon$) = $\exp(-(\varepsilon/k_b T)^2)$. The fluctuating part of the potential function that obeys a Gaussian distribution [5] .

The calculated $\varepsilon$ in Eq. 2 using the experimentally determined diffusion coefficient for the PFV intasome (0.079 ± 0.034 $\mu m^2\ s^{-1}$) yields an energy landscape of 1.1 ± 0.2 $k_B T$ (s.e.). Reducing the diffusion coefficient (0.002 $\mu m^2\ s^{-1}$) to reflect the increased molecular weight of the PFV intasome containing the 13 kb PFV genome, and considering the measured stokes radius of the HIV pre-integration complex (27 nm) [6], we calculate that for a range of stokes radius (20-30 nm)

the free energy landscape will be significantly less than 0.5 $k_BT$. Protein searches on DNA must occur on a timescale that is biologically relevant. Free energy landscapes greater than 2 $k_BT$ are significant enough to limit the utility of a 1D search on DNA. These calculated energy landscapes are clearly less than 2 $k_BT$. We conclude that 1D rotational-coupled translational diffusion is an efficient and biologically relevant search mechanism for the PFV intasome.

**Supplementary References**


1 Einstein, A. The motion of elements suspended in static liquids as claimed in the molecular kinetic theory of heat. *Ann Phys-Berlin* **17**, 549-560, (1905).
2 Maertens, G. N., Hare, S. & Cherepanov, P. The mechanism of retroviral integration from X-ray structures of its key intermediates. *Nature* **468**, 326-329, (2010).
3 Gupta, K. *et al.* Solution conformations of prototype foamy virus integrase and its stable synaptic complex with U5 viral DNA. *Structure* **20**, 1918-1928, (2012).
4 Bagchi, B., Blainey, P. C. & Xie, X. S. Diffusion constant of a nonspecifically bound protein undergoing curvilinear motion along DNA. *J Phys Chem B* **112**, 6282-6284, (2008).
5 Blainey, P. C. *et al.* Nonspecifically bound proteins spin while diffusing along DNA. *Nat Struct Mol Biol* **16**, 1224-1229, (2009).
6 Serrao, E. *et al.* Integrase residues that determine nucleotide preferences at sites of HIV-1 integration: implications for the mechanism of target DNA binding. *Nucleic Acids Research* **42**, 5164-5176, (2014).
